# Innovative 3D-Printed Devices with Modified Prednisolone Release for Colon-Targeted Drug Delivery Systems

**DOI:** 10.3390/pharmaceutics18091185

**Published:** 2026-09-19

**Authors:** Dareena Jaiseri, Supusson Pengnam, Praneet Opanasopit, Theerada Taesotikul, Boonnada Pamornpathomkul, Chaiyakarn Pornpitchanarong, Phuvamin Suriyaamporn, Theerasak Rojanarata, Teeratas Kansom, Thapakorn Charoenying

**Affiliations:** 1 Pharmaceutical Development of Green Innovations Group (PDGIG), Division of Industrial Pharmacy, Faculty of Pharmacy, Silpakorn University, Nakhon Pathom 73000, Thailand; 2 Pharmaceutical Development of Green Innovations Group (PDGIG), Division of Biopharmaceutical Sciences and Pharmacology, Faculty of Pharmacy, Silpakorn University, Nakhon Pathom 73000, Thailand; 3 Research and Innovation Center for Advanced Therapy Medicinal Products, Faculty of Pharmacy, Silpakorn University, Nakhon Pathom 73000, Thailand

**Keywords:** colon-targeted drug delivery system, fused deposition modeling, modified drug release, prednisolone, personalized medicine, three-dimensional printing

## Abstract

**Background**: Prednisolone conventional tablet is a drug commonly used to treat inflammatory bowel disease (IBD). However, the long-term use of high doses of prednisolone can lead to gastric irritation, risk of infection, and other systemic complications. These limitations of the drug can be overcome by using three-dimensional (3D) printing technology with pH-dependent approaches. **Objectives**: This study aimed to develop 3D-printed devices containing prednisolone tablets and gel with an enteric coating for colon-targeted modified release. **Methods**: 3D-printed drug-containing devices (CDDs) were designed and divided into two classifications, including devices for prednisolone tablets (PTDs) and devices for prednisolone gel (PGDs). CDDs were fabricated using fused deposition modeling (FDM). The morphology of the CDDs was assessed using a microscope. The tablets and gel of prednisolone were loaded into CDDs depending on the type of device, and the CDDs were coated by Eudragit^®^ S100. In vitro drug release studies were investigated using dissolution apparatus II in simulated gastric medium (pH 1.2) for 2 h, simulated intestinal medium (pH 6.8) for 2 h, and simulated colonic medium (pH 7.4) for 20 h. **Results**: The prednisolone-loaded CDDs with enteric coating were protected from drug release before reaching the colon. The dissolution profile of prednisolone from CDDs showed no drug release in the simulated gastric and simulated intestinal medium, with drug release initiated under simulated colonic medium and sustained release maintained for up to 24 h. **Conclusions**: These findings showed that CDDs have potential for delivering prednisolone to the colon with controlled drug release under simulated in vitro gastrointestinal conditions. Furthermore, these CDDs could be adapted for other drugs.

## 1. Introduction

Prednisolone is a corticosteroid drug commonly used for the treatment of inflammatory bowel disease (IBD), including Crohn’s disease and ulcerative colitis [1]. The oral administration of prednisolone recommends starting with a high dose of 40 mg daily and decreasing the dosage by 5 mg at weekly intervals, leading to an 8-week treatment period [2,3]. In contrast, the long-term use of prednisolone at doses of 20 mg or higher daily for more than two weeks increases the risk of infection [4]. In addition, the limitations of the drug have many side effects, such as gastric irritation, osteoporosis, and other systemic complications [5]. However, prednisolone is largely absorbed in the upper gastrointestinal tract and readily enters the systemic circulation because it is classified as a Biopharmaceutics classification system (BCS) Class I drug, owing to its high solubility and high permeability [6,7]. Conventional prednisolone tablets release the drug immediately, which may lead to systemic exposure and adverse effects, such as gastric irritation, when used for the treatment of IBD [6,8]. Currently, oral colon-targeted drug delivery systems (CTDDSs) are a strategy to overcome the obstacles of drug delivery to the colon. The systems are designed to enhance the bioavailability, increase the therapeutic efficacy of drugs, direct treatment at the site of the disease, and reduce the side effects of drugs [9,10]. Several approaches have been developed to deliver the drug to the colon, such as the pH-dependent, time-dependent, pressure-controlled, or microbially triggered approaches [11]. The pH-dependent approach uses pH-dependent enteric polymer coatings for drug delivery to the colon [12]. Enteric coatings are extensively used in IBD treatments to delay the release of the drug and increase localized therapeutic effects [13]. However, prednisolone was used at high doses and for long periods in the treatment of IBD, leading to side effects for IBD patients [4]. Recently, three-dimensional (3D) printing technology has been applied to develop personalized medicine for patients, leading to reduced dosage, enhanced treatment effectiveness, and minimized side effects [14,15].

3D printing is an innovative technology that has been widely researched in the pharmaceutical field and is used to personalize the dosage of various medicines. Fused deposition modeling (FDM) is one of the techniques used in 3D printing that fabricates the 3D structure through extrusion deposition methods using filament [15,16]. The benefits of FDM 3D printing include high precision, ease of use, a variety of commercially available filaments, cost-effectiveness, rapid prototyping, and the ability to produce complex geometries [17]. Pharmaceutical applications have been extensively researched using FDM 3D printing to develop 3D-printed tablets by loading drugs into filament and commercial filament that is not directly drug-loaded to create devices to contain the drugs with complex geometric internal and external structures to control the drug release rate [18,19,20,21,22]. However, some drugs are directly loaded into the filament using hot melt extrusion (HME). This method is unsuitable for thermally unstable drugs due to the fact that the HME and FDM techniques have a high temperature process [23]. On the other hand, incorporating the drug directly into the device could avoid the high temperatures of the HME and FDM processes. In addition, it could reduce the preparation process of drug-loaded filaments when compared with commercial filaments [20,24,25]. According to previous research, Skowyra et al. [26] successfully developed the prednisolone-loaded polyvinyl alcohol (PVA) filament for extended-release tablets using FDM 3D printing. The results showed that it is possible to control the tablet weight and drug dose by modifying the model volume. The in vitro drug release of prednisolone was studied using a pH-change flow-through cell dissolution system (pH 1.2 and 6.8). The results showed that all dosages of the 3D-printed prednisolone tablets exhibited extended release. Horváth et al. [27] fabricated the 3D-printed tablets using prednisolone-loaded PVA filament with different settings for the structure of the printlet. The results found that the printlets were successfully fabricated, and the octahedron cube structures reached 100% drug release the fastest after 1.5 h, and other structures showed complete release within 5 h. Sadia et al. [28] fabricated immediate-release prednisolone tablets. The drug-loaded filament was prepared by mixing Eudragit E, prednisolone, and other components using HME. The in vitro release from the printed tablets showed that the drug was released within 30 min. Previous studies have focused on oral dosage forms using prednisolone-loaded filaments to directly fabricate 3D-printed tablets using FDM, but there is a lack of 3D-printed devices incorporating prednisolone with modified release for CTDDS. Therefore, we investigated the application of FDM 3D printing technology to fabricate 3D-printed devices containing prednisolone and combined it with the pH-dependent approach for CTDDS.

Therefore, the aim of this study was to develop a 3D-printed device tablet containing a prednisolone conventional immediate-release tablet and prednisolone gel formulation for modified drug release in the colon. The 3D-printed tablet devices were divided into two classifications, including the device for containing tablets, with different types of polymers and various sizes of holes, and the device for containing gel formulation, with different types of polymers and different numbers of holes. Commercial PVA and polylactic acid (PLA) filaments were used to print the devices. 3D-printed tablet devices were fabricated by FDM, and the drug was incorporated into the devices depending on the type of device. 3D-printed drug-containing devices (CDDs) were coated with Eudragit^®^ S100 for CTDDs. The weight variation, appearance, and morphology of 3D-printed tablet devices were analyzed. The prednisolone dissolution profiles and kinetic release from CDDs were investigated. The novelty of this study is the development of a 3D-printed CTDDS different prednisolone dosage form, including conventional immediate-release tablets and gel formulations, with specifically designed device architecture.

## 2. Materials and Methods

### 2.1. Materials

Prednisolone powder was supported by the Government Pharmaceutical Organization (GPO) (Bangkok, Thailand). Prednisolone conventional immediate-release tablet (5 mg/tablet; product name: Prednisolone (White); batch No. AP19) manufactured by Medicine Products Co., Ltd. was purchased from Medicine Products Co., Ltd. (Nakhonpathom, Thailand). Polyvinylpyrrolidone (PVP) with a molecular weight of ~1,300,000, sodium phosphate dibasic heptahydrate (Na_2_HPO_4_ · 7 H_2_O), potassium phosphate monobasic (KH_2_PO_4_), Eudragit^®^ S100, and sodium chloride (NaCl) were purchased from Sigma-Aldrich (Steinheim, Germany). Potassium chloride (KCl) was purchased from QReC™ (Auckland, New Zealand). Hydrochloric acid fuming 37% (HCl) and sodium hydroxide (NaOH) were provided by Merck KGaA (Darmstadt, Germany). PVA and PLA filament (1.75 mm) were purchased from Shenzhen eSun Industrial Co., Ltd., (Shenzhen, China). All other chemicals used in this experiment were of analytical grade.

### 2.2. CDDs Designed and Fabricated

The CDD model was sketched using Autodesk^®^ Fusion 360^TM^ Student software (v.2.0.7819) (Autodesk, Inc., San Francisco, CA, USA), and then the slicer program was used for generating the G-code for the 3D printer. An FDM 3D printer (Prusa i3 MK3, Prusa Research S.R.O., Prague, Czech Republic) was used to fabricate the CDDs. The commercial PLA and PVA filaments were used for 3D printing and were selected to print the CDDs. The parameters for setting 3D printing were presented in Table 1. The CDDs were designed to be divided into two classifications, composed of devices for prednisolone tablets (PTDs) and devices for prednisolone gel (PGD).

### 2.3. Designed and Fabricated PTDs

**Figure 1:**
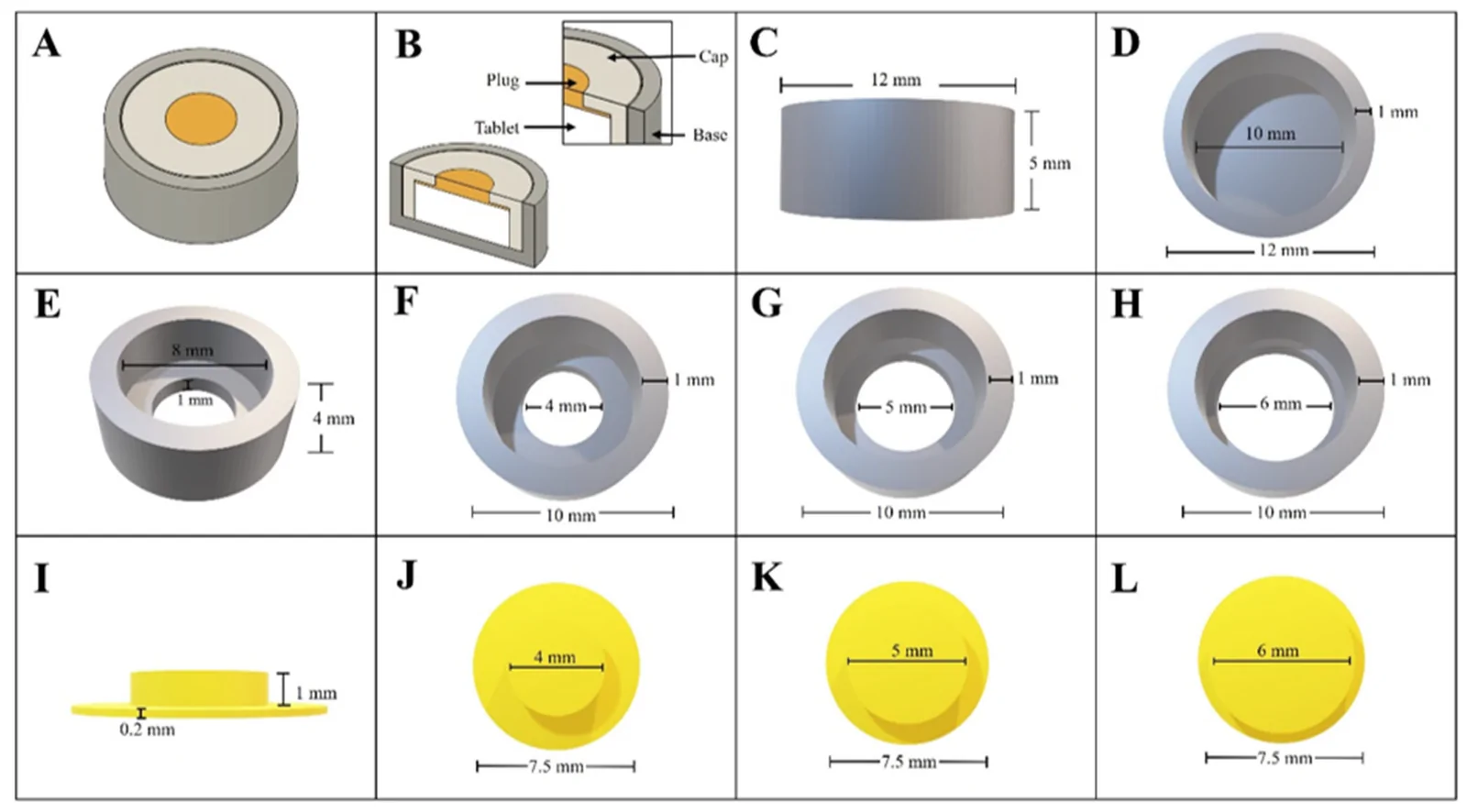
The dimensions of PTDs: (**A**) the entirety of PTDs, (**B**) the vertical cross-section of the PTDs, (**C**,**D**) the base, (**E**) the cap, (**F**–**H**) the upper section of the cap with different sizes of holes (4, 5, and 6 mm), (**I**) the front section of the plug, and (**J**–**L**) the upper section of the plug with different sizes (4, 5, and 6 mm).

The 3D-printed PTDs were designed to contain prednisolone tablets and to provide modified drug release. The PTDs were designed to consist of three parts, including a base, cap, and plug, with different hole sizes (4, 5, and 6 mm). The dimensions of the PTDs are presented in Figure 1. The fabricated PTDs were classified into two types: the devices printed using PLA filament for the base and cap with PVA filament for the plug (PTDs-LV), and the devices printed entirely using PVA filament for the base, cap, and plug (PTDs-V).

### 2.4. Designed and Fabricated PGDs

**Figure 2:**
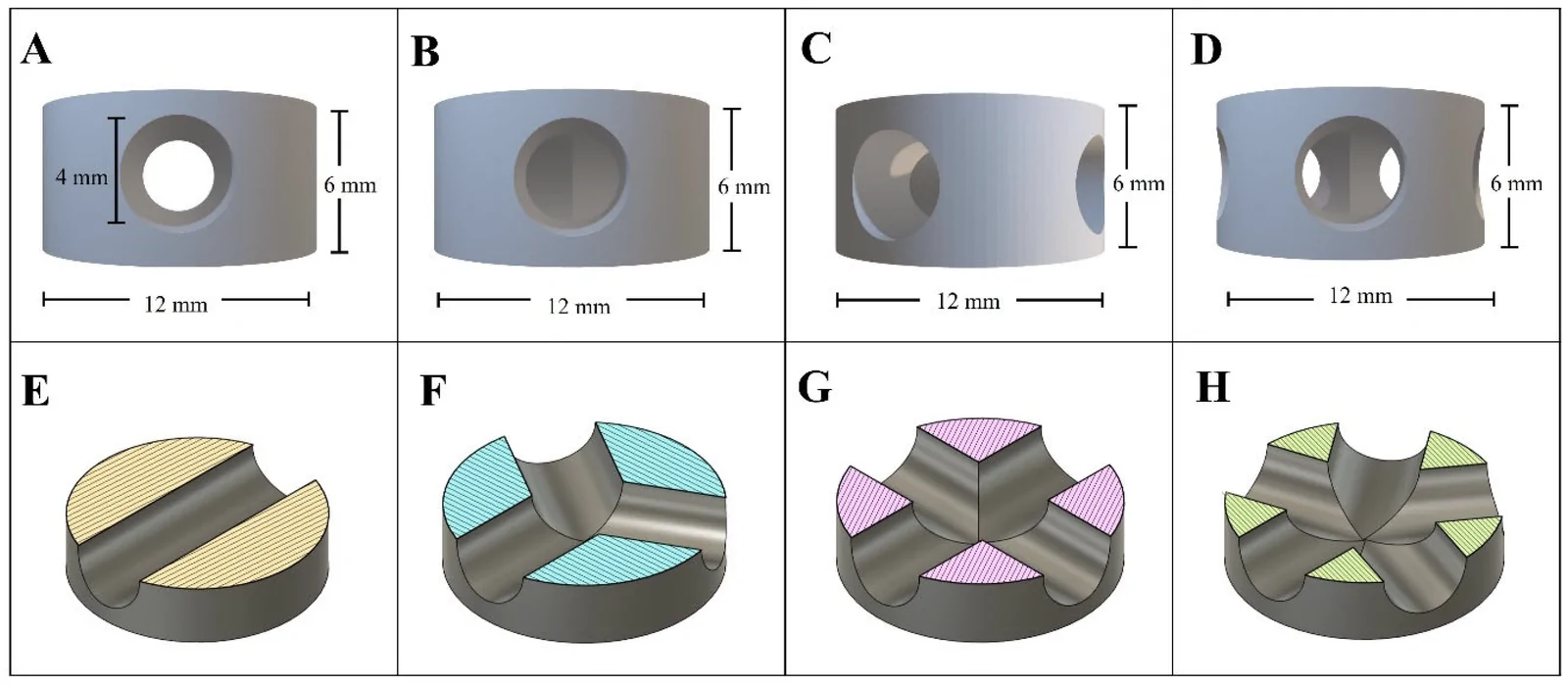
The dimensions of PGDs: (**A**–**D**) the dimensions of PGDs with 2, 3, 4, and 5 holes, respectively, and (**E**–**H**) the internal structure showed by a horizontal cross-section of the PGDs to illustrate the hole pattern (2, 3, 4, and 5 holes).

The PGDs were designed in a tablet shape, with a diameter of 12 mm and a height of 6 mm. To accommodate the drug gel, the PGDs were designed with varying numbers of holes (2, 3, 4, and 5) on the lateral sides of the devices, with each hole having a radius of 4 mm. These holes were created to contain the prednisolone gel and to modulate the drug release rate. The dimensions of the PGDs are illustrated in Figure 2. The fabricated PGDs were classified into two types: the devices produced using PLA filament (PGDs-L) and the devices produced using PVA filament (PGDs-V).

### 2.5. Characterization of CDDs

The appearance of the CDDs was observed under a digital microscope. In addition, a scanning electron microscope (SEM) (Tescan Mira 3, Brno, Czech Republic) was used to evaluate the surface morphology of the CDDs. The weight variation in the CDDs was measured using an analytical balance by weighing 20 individual pieces from each part. The actual size (height, diameter, and width of hole) of the CDDs was assessed using a digital Vernier caliper (Zhejiang Deqing Syntek Electronic Technology CO., Ltd., Huzhou, Zhejiang, China). The data were presented as mean ± standard deviation (SD) (*n* = 20).

### 2.6. Incorporating the Drug into the CDDs

The prednisolone conventional immediate-release tablet was placed in the PTDs. The PGDs were loaded with prednisolone gel. The preparation of the prednisolone gel was composed of prednisolone powder, PVP, and water at the weight ratio of 0.5:2.5:5, respectively. Then, the prednisolone gel was treated to remove air bubbles by placing the prednisolone gel into the vacuum chamber for 2 h. Next, the prednisolone gel was loaded within the empty hole of the PGDs using a syringe; the weight of the prednisolone gel filled into the devices was 140, 200, 240, and 280 mg (corresponding to prednisolone doses of 8.75, 12.50, 15.00, and 17.50 mg, respectively) for PGDs with the 2, 3, 4, and 5 holes, respectively. A digital balance was used to determine the weight of the loaded prednisolone gel. After that, the prednisolone gel-loaded PGDs was kept in a hot air oven at 50 °C for 2 h to remove the moisture from the gel.

### 2.7. Analytical Performance Evaluation of UV Spectrophotometric Method

The analytical performance of the UV spectrophotometric method (VICTOR^®^ Nivo™ multimode plate readers, PerkinElmer, Waltham, MA, USA) was adapted from the validated method reported by Nasereddin et al. [29]. The prednisolone quantification method was evaluated in terms of specificity, linearity, accuracy, and precision at a wavelength of 246 nm. The specificity of the method was assessed by evaluating potential interference from formulation components and device materials. The linearity of the analytical method was evaluated by generating a calibration curve using prednisolone standard solutions prepared in simulated colonic medium (pH 7.4) over the concentration range of 1.87–40 µg/mL. The absorbance of each standard solution was measured in triplicate, and the mean values were used to generate the calibration curve. The accuracy of the method was assessed by recovery testing at three concentration levels (15, 30, and 40 µg/mL). Each concentration level was analyzed in triplicate, and the percentage recovery was calculated by comparing the measured prednisolone concentration with the theoretical concentration. The precision of the analytical method was evaluated by replicate measurements of prednisolone standard solutions at three concentration levels (15, 30, and 40 µg/mL) and expressed as percentage relative standard deviation (%RSD).

### 2.8. Enteric Coating of CDDs

The enteric coating enabled delivery of the devices to the colon while preventing premature drug release. The enteric solution was prepared by mixing 100 mL of isopropanol with 100 mL of acetone (*v*/*v*), and then 20 g of Eudragit^®^ S100 was dissolved in the solution. The CDDs were coated with an enteric solution using the dip coating technique. Then, the enteric-coated devices were dried in a hood for 30 min, and the process was repeated for two cycles. After that, the enteric-coated devices were placed in a hood to dry overnight at room temperature. The weight of the enteric coating was determined by subtracting the weight before coating from the weight after the final drying. The coating thickness was evaluated from SEM images of three enteric-coated of PGDs. For each device, the coating thickness was measured at three locations, and the average coating thickness was calculated. The mean thickness value of the three devices was then reported as mean ± SD.

### 2.9. Drug Content Uniformity and Drug Loss Evaluation of Prednisolone Gel-Loaded PGDs

The prednisolone content was determined before and after the gel preparation process to evaluate drug loss during gel handling and drying. The prednisolone content was quantified using the UV spectrophotometric method at 246 nm [29]. The content uniformity was evaluated based on the %RSD of prednisolone content of ten individual PGDs-L with 2 holes (*n* = 10). For drug content determination, the prednisolone gel was separated from PGD-L by dissolving in 900 mL of simulated colonic medium (pH 7.4) using a bath sonicator at 37 °C. Complete drug release was considered to have been achieved when no appreciable further increase in prednisolone concentration was observed between consecutive sampling points. The percentage of drug loss was calculated as the percentage difference between the initial and final prednisolone content.

### 2.10. In Vitro Dissolution Study

The dissolution profiles of the prednisolone conventional immediate-release tablet (control), prednisolone tablet-incorporated PTDs with an enteric coating, and PGD-loaded prednisolone gel with an enteric coating were evaluated using a USP dissolution apparatus II (DT 720, ERWEKA GmbH, Heusenstamm, Germany) with a paddle speed fixed at 75 rpm, and the experiments were performed at 37 ± 0.5 °C. The dissolution testing was selected to simulate the physiological transit time and pH gradient along the human gastrointestinal tract [9,30]. The medium consisted of 900 mL, with simulated gastric medium (pH 1.2) for 2 h, simulated intestinal medium (pH 6.8) for 2 h, and simulated colonic medium (pH 7.4) for 20 h. The medium was sampled by withdrawing 3 mL at various time points (15, 30, 45 min, 1, 2, 3, 4, 5, 6, 8, 10, 12, and 24 h), and 3 mL of fresh medium was added to replace the withdrawn sample. The sample of the medium was filtered through a 0.45 μm nylon filter. The percentage of prednisolone release was determined using a UV spectrophotometer at a wavelength of 246 nm [29].

### 2.11. Kinetic of Drug Release from CDDs

The kinetics of drug release from the prednisolone tablet-loaded PTDs and prednisolone gel-loaded PGDs were assessed using the first-order, zero-order, and Higuchi kinetics models. The kinetic analysis was performed using the drug release data obtained during the active release phase, from the onset of measurable drug release until complete drug release was achieved. The result was evaluated by calculating the coefficient of determination (R^2^) to evaluate the best-fit kinetic model of the equations with the drug release profile.

### 2.12. Statistical Analysis

The experimental results were shown as the mean ± SD of triplicate samples. Statistical analysis was performed using a two-way analysis of variance (ANOVA) to evaluate the effects of device geometry and dissolution time on the release behavior of CDDs, followed by Dunnett’s multiple comparisons test. The effects of hole size in PTDs and the number of holes in PGDs were evaluated at corresponding dissolution time points. Statistical significance was set at *p* < 0.05.

## 3. Results and Discussion

### 3.1. Characterization of CDDs

**Figure 3:**
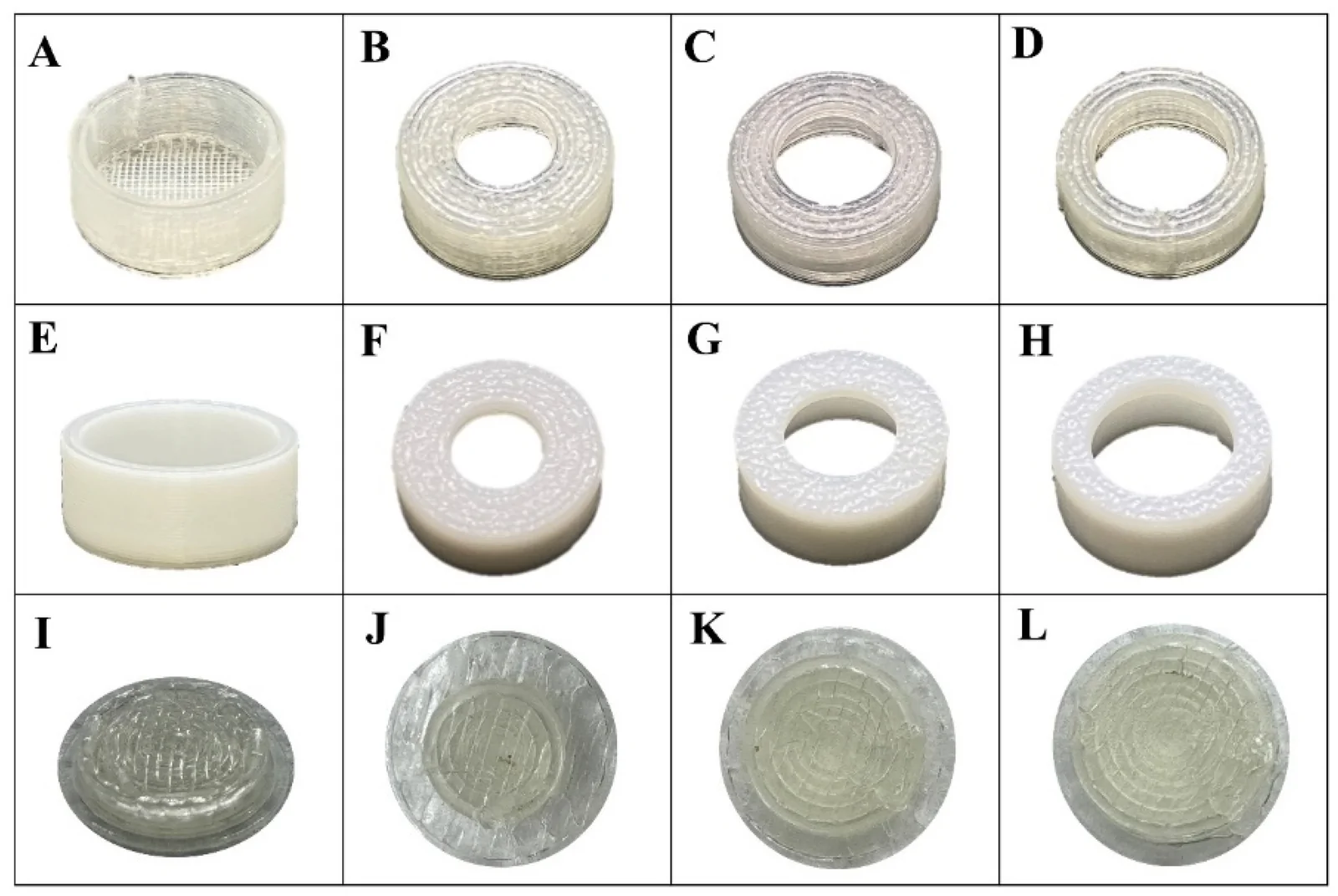
The PTDs of each part: (**A**) the base part with PVA; (**B**–**D**) the cap part for PVA with different sizes of holes (4, 5, and 6 mm), respectively; (**E**) the base part with PLA; (**F**–**H**) the cap part for PLA with different sizes of holes (4, 5, and 6 mm), respectively; (**I**) the front section of the plug part with PVA; (**J**–**L**) the upper section of the plug for PVA with different sizes (4, 5, and 6 mm), respectively.

**Figure 4:**
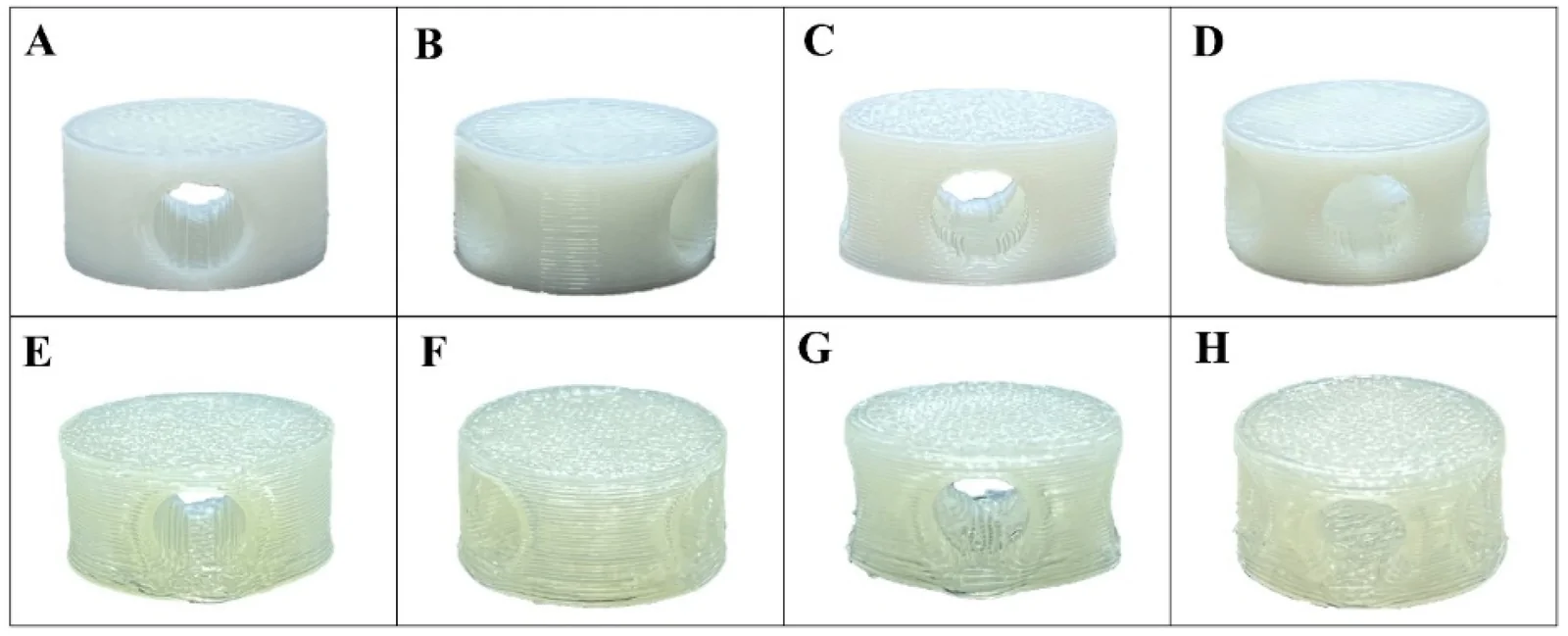
The PGDs; (**A**–**D**) PGDs-L with different numbers of holes (2, 3, 4, and 5 holes, respectively) and (**E**–**H**) PGDs-V with 2, 3, 4, and 5 holes, respectively.

**Figure 5:**
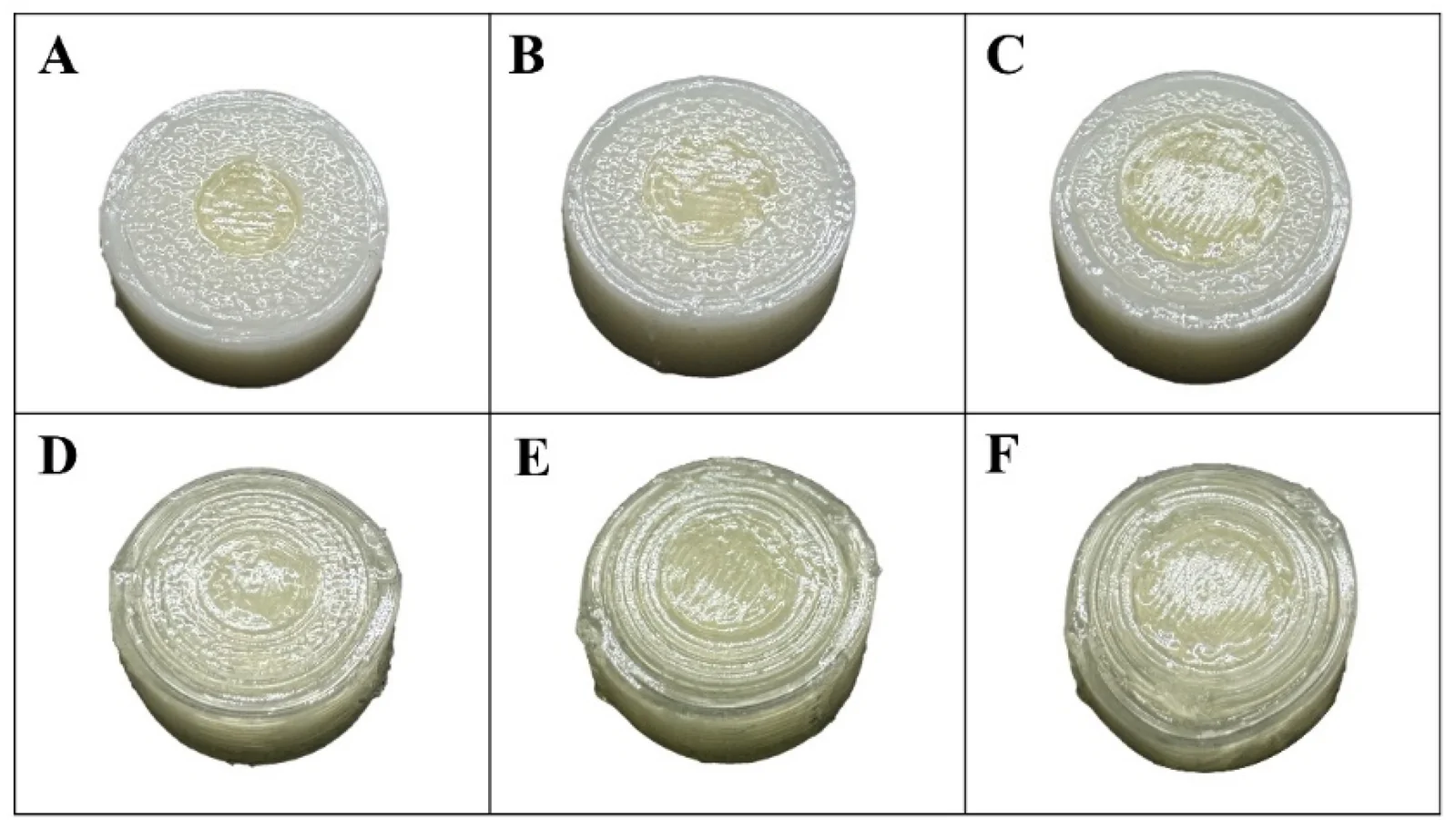
The prednisolone tablet-loaded PTDs with enteric coating; (**A**–**C**) PTDs-LV with different sizes of the holes (4, 5, and 6 mm), respectively, and (**D**–**F**) PTDs-V with different sizes of the holes (4, 5, and 6 mm), respectively.

**Figure 6:**
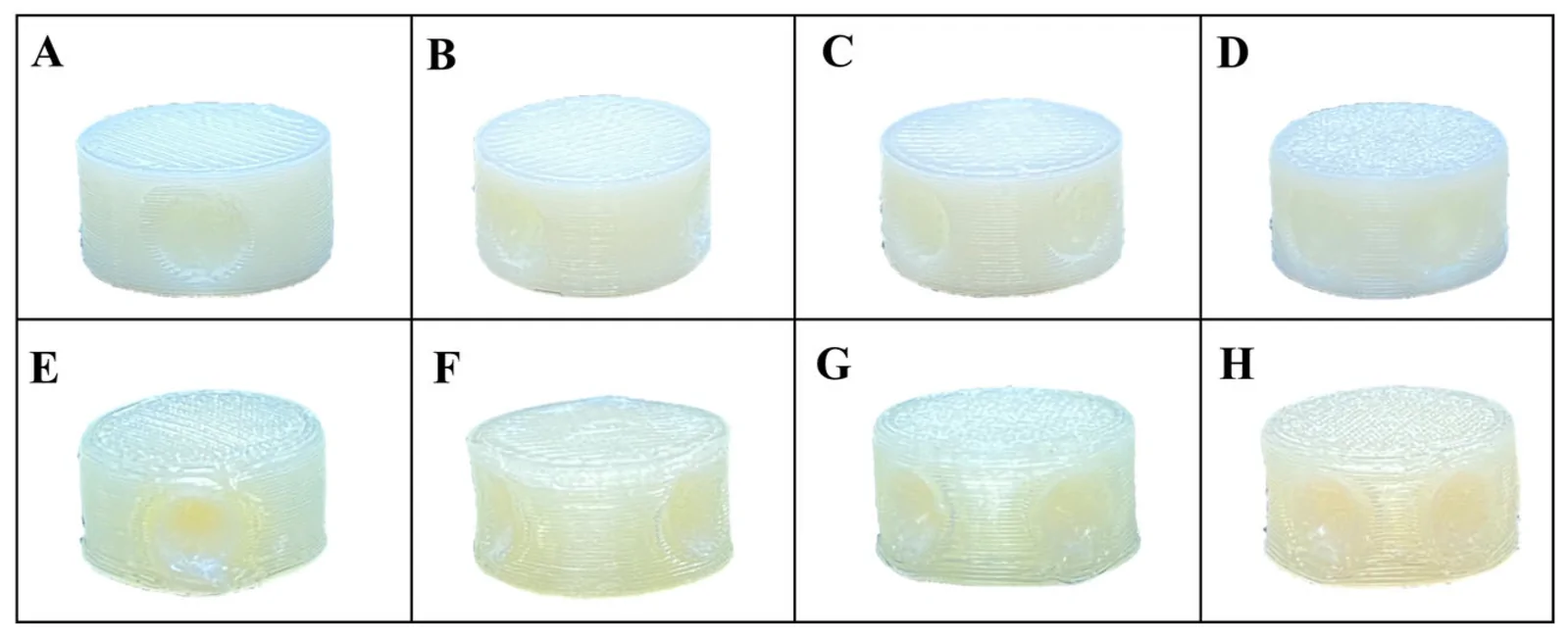
The prednisolone gel-loaded PGDs with enteric coating; (**A**–**D**) PGDs-L with varying numbers of holes (2, 3, 4, and 5 holes, respectively) and (**E**–**H**) PGDs-V with varying numbers of holes (2, 3, 4, and 5 holes, respectively).

**Figure 7:**
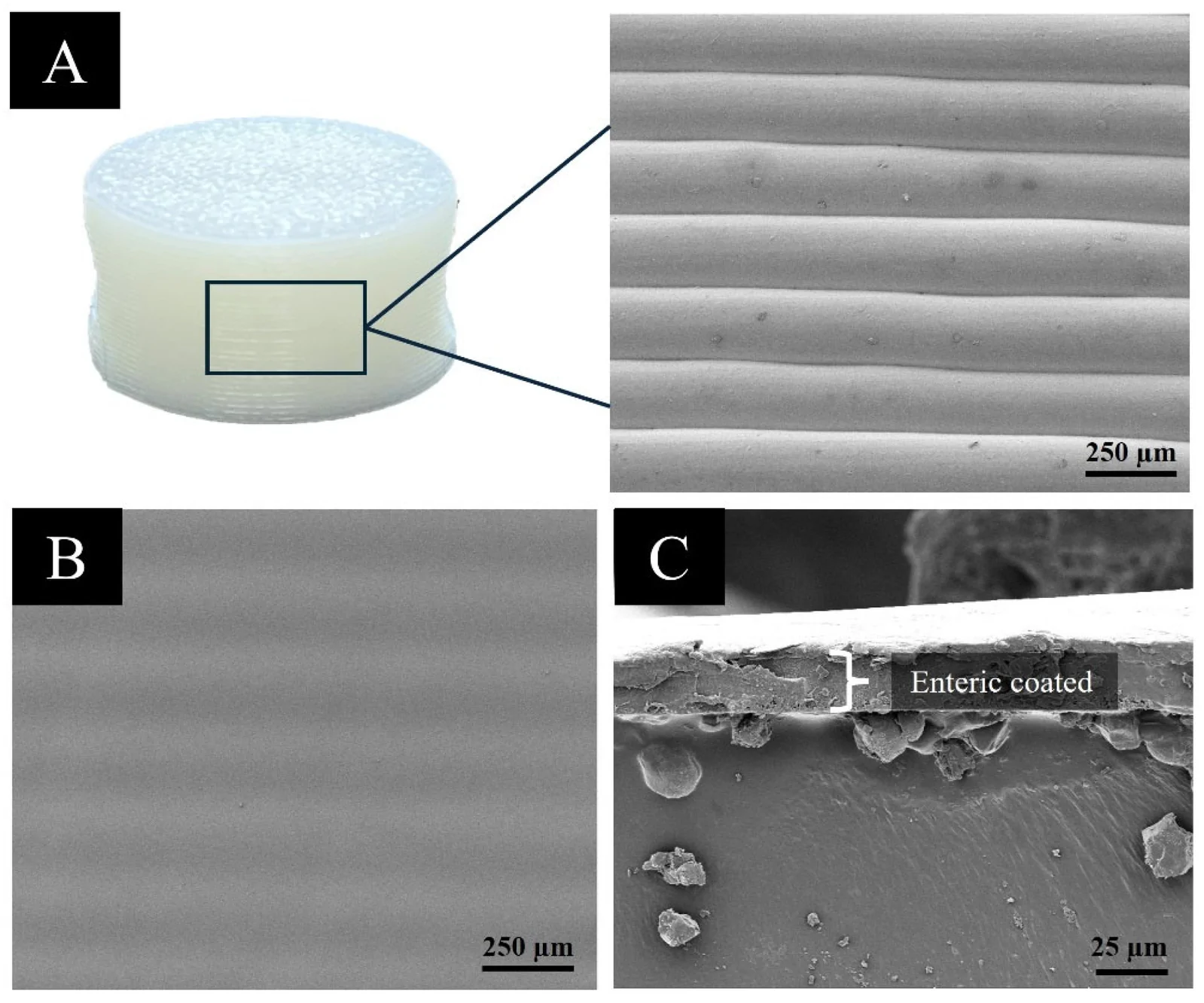
SEM image of the PGDs; (**A**) the surface before coating (120×), (**B**) the surface after coating (120×), (**C**) a coating thickness (1000×).

The CDDs were successfully fabricated using an FDM 3D printer with commercial filaments (PVA and PLA). The appearance of PTDs and PGDs was analyzed using a digital microscope, and the obtained images were presented in Figure 3 and Figure 4, respectively. The white color represents PLA filament, the light beige color indicates PVA filament, and the CDDs retained the same color as the original filament. This layer structure of the CDDs was obtained due to the process of FDM 3D printing, which extruded the material and printed the device layer by layer [19,31]. The printed CDDs had dimensions consistent with the design, showing the accuracy and reliability of the FDM 3D printer [32]. The weight and dimensions of the CDDs are shown in Table 2 and Table 3. The low SD value of the CDDs confirmed the uniformity in size and shape. The findings were consistent with a previous article, which reports that the FDM 3D printer has the highest precision, achieving a low weight error of 0.05% [31,33]. The PTDs with varying sizes of the holes in the cap component and PGDs with varying numbers of holes were a crucial factor in controlling drug release. The low SD of the hole sizes indicated that variations in drug release due to inaccuracies in hole dimensions of the CDDs were unlikely. The prednisolone tablet-loaded PTDs and prednisolone gel-loaded PGDs with an enteric coating are illustrated in Figure 5 and Figure 6, respectively. The surface of the CDDs was smooth, and the SEM image exposed a simple stripe pattern (Figure 7A). The surface of the coated devices was smooth. The average coating thickness determined from SEM images of the devices was 21.54 ± 0.41 µm (Figure 7B,C). The weight variation in the prednisolone-loaded CDDs before and after enteric coating, as well as the calculated weight of the enteric coating, are reported in Table 4. The low SD of the coating thickness and enteric-coated weight demonstrated excellent coating uniformity of CDDs in each tablet, which is essential for precise and reproducible CTDDS [34,35].

### 3.2. Analytical Performance Evaluation of UV Spectrophotometric Method for Prednisolone Quantification

The analytical performance of the UV spectrophotometric method for prednisolone quantification was evaluated before dissolution testing. The specificity of the UV spectrophotometric method was evaluated to confirm that formulation components and device materials did not interfere with prednisolone quantification at 246 nm. The UV wavelength scanning spectra of prednisolone, PVP, PVA derived from filament, Eudragit^®^ S100, and simulated colonic medium (pH 7.4) were evaluated using concentrations corresponding to the highest amounts of each component in the formulations (Supplementary Figures S1–S5). These individual components showed no interfering absorbance at 246 nm. Furthermore, the potential interference of PVP, PVA derived from filament, and Eudragit^®^ S100 in simulated colonic medium (pH 7.4) on prednisolone quantification was evaluated. The measured absorbance values were comparable to those of prednisolone solutions prepared in simulated colonic medium (pH 7.4) without these components, indicating that these components did not interfere with prednisolone quantification at 246 nm. Therefore, the tested medium was considered suitable as a blank for prednisolone quantification. The analytical performance parameters are summarized in Table 5. The method showed excellent linearity over the concentration range of 1.87–40 µg/mL, with a high R^2^ = 0.9997 (Supplementary Figure S6). The accuracy of the method was evaluated by recovery testing, with recovery values ranging from 99.40% to 100.38%, indicating acceptable accuracy, while the low %RSD values (0.46–1.00%) confirmed good precision and repeatability of the analytical method. These findings are consistent with previous reports on UV spectrophotometric determination of prednisolone, where the method demonstrated good linearity, acceptable accuracy, and precision for quantitative analysis of prednisolone in pharmaceutical formulations [29].

### 3.3. Drug Content Uniformity and Drug Loss Evaluation of Prednisolone Gel-Loaded PGDs

The prednisolone content of PGD-loaded prednisolone gel showed low variability (%RSD = 0.09), indicating good reproducibility of the gel loading process. The effect of the preparation process on prednisolone content was further evaluated by comparing the drug content before and after drying at 50 °C. The percentage of drug loss was only 0.12 ± 0.10%, suggesting that neither gel loading nor drying caused appreciable loss of prednisolone. Overall, these results indicate that the preparation conditions were suitable for maintaining consistent prednisolone content in the PGDs.

The low variability in prednisolone content in PGD-loaded prednisolone gel indicates that the developed gel loading process provided consistent drug incorporation within the devices. This reproducibility is important for ensuring consistent drug incorporation during gel loading. Furthermore, the minimal drug loss observed after drying at 50 °C suggested that the selected processing condition was appropriate for maintaining prednisolone content. Prednisolone has been reported to exhibit relatively high thermal resistance in the solid state, with melting transitions occurring at approximately 220–249 °C [36]. Therefore, the drying temperature applied in this study was substantially lower than the reported melting temperature, supporting the suitability of the selected drying condition for preparing the prednisolone-loaded PGDs.

### 3.4. In Vitro Dissolution Study of the CDDs

**Figure 8:**
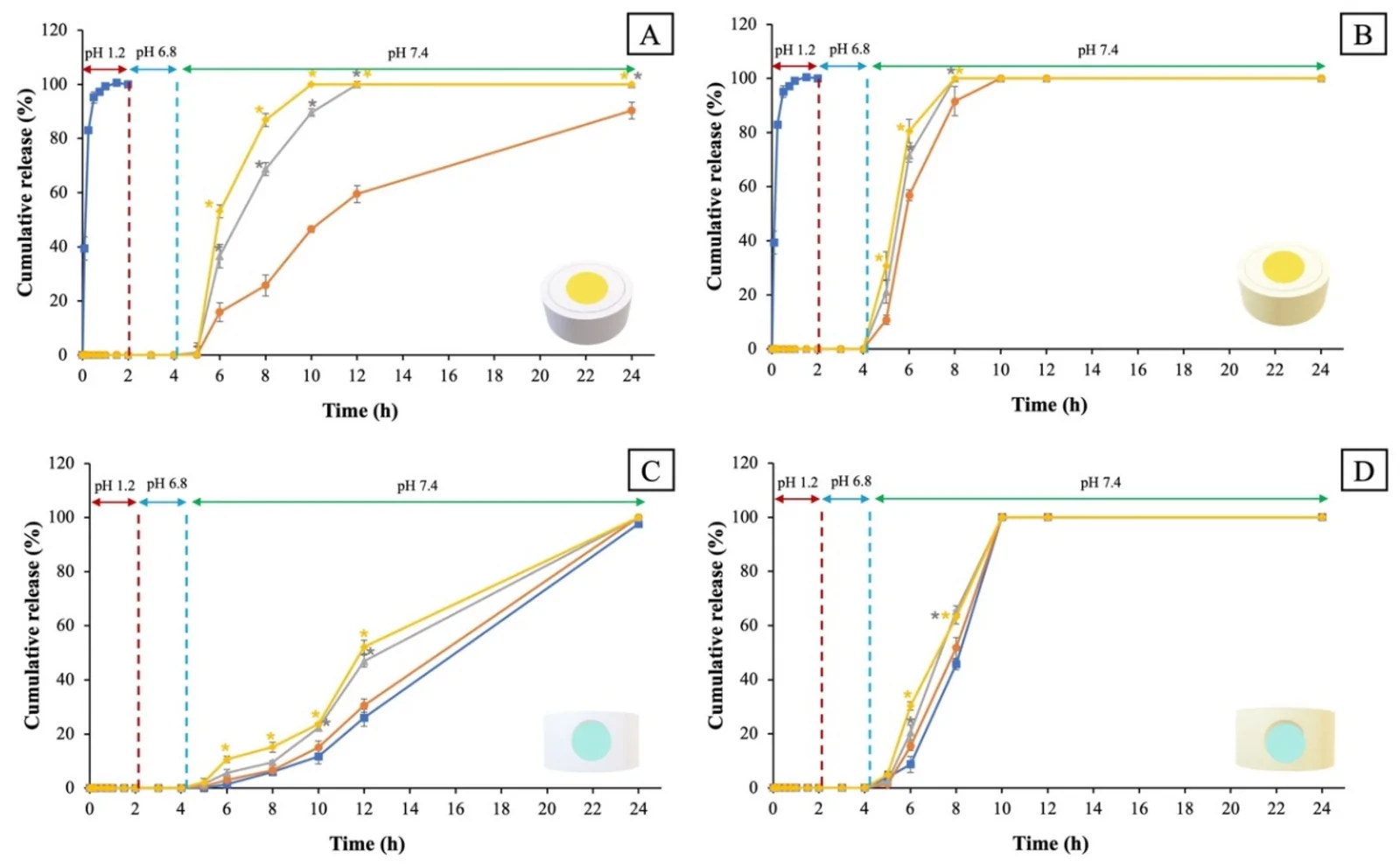
The dissolution profiles of prednisolone-loaded CDDs; (**A**) the PTDs-LV, (**B**) the PTDs-V, (**A**,**B**) a prednisolone conventional immediate-release tablet (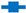), the size of holes, 4 mm (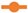), 5 mm (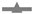), and 6 mm (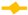), (**C**) the PGDs-L, (**D**) the PGDs-V, (**C**,**D**) the number of holes, 2 holes (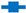), 3 holes (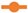), 4 holes (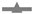), and 5 holes (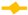). * Statistically significant (*p* < 0.05) compared with the size of holes 4 mm (**A**,**B**), and the number of 2 holes (**C**,**D**).

Prednisolone release studies from the CDDs were conducted to improve the treatment of IBD, enable dose adjustment to meet individual patient needs, reduce dosing frequency, and minimize adverse effects. The results show that the prednisolone conventional immediate-release tablet exhibited an immediate-release profile in the simulated gastric medium (pH 1.2), achieving complete drug release (100%) within 30 min (Figure 8A,B). This result is consistent with a previous article reporting the immediate release of the prednisolone conventional immediate-release tablet [6]. The prednisolone conventional immediate-release tablet was incorporated into the PTDs, and the prednisolone gel formulation was contained in the PGDs. The results demonstrated that the enteric-coated CDDs protected the drug from premature release under simulated gastric medium and intestinal medium and showed drug release under simulated colonic medium (Figure 8). The drug release in the simulated colonic medium at pH 7.4 was due to the coating using the pH-dependent enteric polymer (Eudragit^®^ S100), which dissolves above pH 7 [37]. The pH dependence of the present system is related to the concept of stimulus-responsive drug delivery. In this study, Eudragit^®^ S100 functions as a pH-dependent enteric polymer coating that prevents premature drug release and controls drug release under simulated colonic medium. This mechanism is consistent with the principle of stimulus-responsive delivery systems, where environmental changes are used to regulate drug release [38]. Recent developments in smart materials and advanced fabrication approaches, including stimulus-responsive materials and four-dimensional (4D) printing technologies, have provided additional strategies for developing adaptive drug delivery systems [38,39]. However, these complex technical approaches are non-essential to achieve the objectives of the present study. This study utilizes a simple and standard approach using a pH-dependent enteric polymer through Eudragit^®^ S100 to achieve colon-targeted release under simulated in vitro gastrointestinal conditions. Therefore, these CDDs provide a simple method for pH-dependent enteric polymer drug release without using complex mechanisms or multiple triggers.

The dissolution performed for PTDs-LV with the size of holes 4, 5, and 6 mm exhibited the sustained release profiles in the simulated colonic medium (pH 7.4). The PTDs-LV with the size of holes 4 mm showed a slower release rate, achieving approximately 15% and 60% cumulative release at 6 and 12 h, respectively, and more than 90% after 24 h, while the size of holes 5 and 6 mm demonstrated a rapid release profile when compared with the size of holes 4 mm and completed cumulative release of more than 90% within 10 h (Figure 8A). The release behavior from PTDs-V with varying hole sizes was slow, and cumulative release reached more than 90% in 8 h (Figure 8B).

Figure 8C shows the drug release characteristics from PGDs-L with varying numbers of holes. The results showed that the two and three holes exhibited a slower release profile, releasing about 25% at 12 h and achieving cumulative release within 24 h, whereas the four and five holes demonstrated a rapid release rate, releasing around 45% after 12 h and completing release at 24 h. The PGDs-V demonstrated a slow-release pattern. The PGDs-V with the number of holes 2 and 3 were obtained approximately 45% after 8 h. In comparison, those with four or five holes showed drug release around 65% after 8 h, and all numbers of holes exhibited 100% cumulative release within 24 h, as shown in Figure 8D. Overall, the cumulative release profiles showed that the release duration of CDDs varied with device geometry. Devices with smaller hole sizes or a lower number of holes exhibited more prolonged drug release, whereas a larger opening area was associated with faster drug release. These findings suggest that geometric parameters may be useful for modulating prednisolone release from the CDDs. However, in the PGDs, increasing the number of holes was also associated with differences in gel amount, drug loading, internal geometry, and diffusion area. Therefore, the observed release behavior reflects the combined effects of these factors, and the contribution of hole number alone cannot be isolated from the present experimental design.

These findings suggest that the release behavior of PTDs is strongly influenced by both the polymer and the geometrical design [33]. In the case of PTDs-LV, the incorporation of PLA and PVA showed a critical role in controlling drug release. The PTDs-LV is designed with a central cavity containing the drug, which is sealed by a PVA plug, while the entire structure is encapsulated by a PLA. Importantly, PLA remains intact with medium due to its hydrophobic polymer, whereas the PVA plug dissolves rapidly upon contact with the dissolution medium [31,40]. Consequently, drug release is initiated by the dissolution of the PVA plug, followed by penetration of the medium through the hole opening, as the surrounding PLA shell does not permit significant medium penetration. In terms of drug release rate, the PTDs-LV exhibit a release behavior highly dependent on the hole diameter. A smaller hole size (4 mm) limits the exposed surface area of the PVA plug to the medium, thereby slowing down its dissolution and delaying the penetration of the medium into the inner cavity. In addition, the structural integrity provided by the PLA enables controlled drug release, resulting in a sustained release profile. On the other hand, PTDs-V composed entirely of PVA exhibit hydrophilic characteristics, allowing the medium to penetrate from all directions, not only through the central hole but also across the entire surface area of the PTDs-V. As a result, PTDs-V do not effectively control the drug release rate, as rapid swelling and dissolution of the matrix lead to a loss of structural stability.

This result was consistent with a previous article, which reported the designed variation in hole size over drug-releasing time [41,42]. Sadia et al. fabricated a 3D-printed caplet by varying the channel size and number of channels. The results found that the larger size of channels and the larger number of channels were important for improving drug release by increasing the surface area of the tablet in contact with the drug and medium [43]. Additionally, the filament material used to print CDDs was crucial to the rate of the drug release. Therefore, the results of this study showed that the use of PVA filament affected the ability to control drug release, resulting in a faster release compared with devices fabricated using PLA filament. Previously, Horváth et al. [27] successfully used prednisolone-loaded PVA filament to fabricate 3D-printed tablets. However, the 3D-printed tablets released the drug in a simulated gastric medium and achieved complete drug release within 5 h. Moreover, the present study was the first to employ conventional prednisolone tablets and prednisolone gel encapsulated within CDDs for CTDDSs, thereby modifying the release profile from rapid to sustained release. This approach was simple to prepare and avoided the high processing temperatures associated with HME, which may lead to drug degradation when the drug is incorporated directly into the filament. In addition, encapsulating prednisolone gel within the PGDs provided flexibility in tailoring the drug dosage to meet individual patient needs. However, the device size was larger than conventional dosage forms, although its dimensions were within the size range described in FDA guidance for solid oral dosage forms. Importantly, device dimensions alone are insufficient to predict swallowability, and the swallowability of the developed CDDs therefore remains to be evaluated in future studies. Furthermore, the PLA-based CDDs are expected to transit through the gastrointestinal tract and be eliminated via fecal excretion [44].

### 3.5. Kinetics of Drug Release from the CDDs

The drug release kinetics of prednisolone-loaded CDDs were determined by fitting the release profiles to zero-order, first-order, and Higuchi models. The kinetic analysis was performed based on the release phase representing the actual dissolution behavior of each formulation, following previously reported approaches for dissolution profile [45,46,47]. This approach allowed the kinetic models to appropriately describe and predict the observed prednisolone release profiles during the active release period. The resulting R^2^ values of the release kinetics are presented in Table 6. The PTDs-LV containing prednisolone tablets with a hole size of 4 mm were evaluated from 5 to 24 h, as complete drug release was achieved within 24 h. In contrast, PTDs-LV with larger hole sizes of 5 and 6 mm showed a shorter active release phase, and active release phase was observed from 5 to 10 h. PTDs-V containing prednisolone tablets with hole sizes of 4, 5, and 6 mm were evaluated from 5 to 8 h, representing the active release phase. The release profiles of the PTDs showed the best fit to the first-order kinetic model compared with the zero-order and Higuchi models, as indicated by the highest R^2^ values. For PGDs-L containing prednisolone gel, kinetic analysis was performed using release data from 5 to 24 h, corresponding to the active release phase until complete drug release was achieved. In contrast, PGDs-V exhibited a measurable drug release interval from 5 to 10 h.

The kinetic model results demonstrated that the PGDs-L were best fitted by the zero-order kinetic model, with the highest R^2^ values among the evaluated models. Similarly, PGDs-V with two and three holes showed the best fit to the zero-order kinetic model. The fitting results of the zero-order kinetic model suggested a relatively constant prednisolone gel release profile during the investigated period, which may be associated with the controlled release effect of the device structure and number of holes [48]. However, PGDs-V with four and five holes exhibited comparable R^2^ values between the Higuchi and zero-order models, with only minor differences observed between the two models. Therefore, the release behavior of these devices could not be distinctly assigned to a specific kinetic model based solely on the R^2^ values. The kinetic analysis was considered as a mathematical approach to describe and observe release profiles rather than to confirm the underlying release mechanism [49,50]. This difference may be related to the increased number of holes in the PVA-based device. The greater orifice area could enhance contact between the prednisolone gel and the dissolution medium, facilitating drug release from the device. As a result, the better fitting of the Higuchi model may reflect the characteristics of the observed release profile rather than confirm a diffusion-controlled release mechanism [51,52].

The differences in release kinetics between the PTDs and PGDs can be attributed to both the physical form of prednisolone and the structural design of the devices. The PTDs containing solid prednisolone tablets followed first-order release kinetics, suggesting that the drug release rate was dependent on the remaining drug concentration within the device. This behavior was characteristic of tablet-based systems, where drug dissolution and diffusion through the release orifice were governed by concentration gradients. As the amount of drug decreased over time, the release rate gradually declined, resulting in concentration-dependent kinetics [53,54].

In contrast, the PGDs-L and PGDs-V with two and three holes containing prednisolone gel exhibited zero-order release kinetics, suggesting a constant drug release rate over time. This release behavior can be explained by the gel matrix acting as a drug reservoir, in which diffusion occurred at a controlled and steady rate through the device openings. The semi-solid nature of the gel minimized concentration-driven fluctuations in release and enabled time-dependent drug delivery independent of drug concentration. Furthermore, the device geometry and number of holes played a crucial role in regulating the diffusion pathway, thereby maintaining a consistent release profile. According to previous research, Jaiseri et al. [55] fabricated PLA floating devices for incorporating propranolol gel; the release kinetics fit well with zero-order kinetics. The findings in this study were consistent with those reported in previous studies on 3D-printed tablets with zero-order release profiles. However, PGDs-V with four and five holes showed comparable fitting between the Higuchi and zero-order models, with only minor differences in the R^2^ values. These results suggest that the kinetic models provided similar descriptions of the observed release profiles, and the model fitting should be interpreted as a predictive approach rather than direct confirmation of the release mechanism.

## 4. Conclusions

In this research, we have successfully developed CDDs using FDM 3D printing technology by conveniently fabricating them with various geometric structures and polymer types (PLA and PVA). The prednisolone conventional immediate-release tablet was incorporated into the PTDs, and the prednisolone gel was loaded into the PGDs. The CDDs were coated with Eudragit^®^ S100 using the dip coating technique for CTDDS. The dissolution profile of prednisolone from CDDs demonstrated delayed release in the simulated gastrointestinal medium, with a sustained release characteristic observed in the simulated colonic medium. The drug release kinetics of PTDs were best described by the first-order model, PGDs-L and PGDs-V with two and three holes showed a better fit with the zero-order model, while PGDs-V with four and five holes exhibited comparable fitting between the Higuchi model. These results demonstrated the potential of CDDs for colon-targeted prednisolone delivery under simulated in vitro gastrointestinal conditions. The combination of drug formulation and 3D-printed device architecture provided a flexible approach for controlling prednisolone release profiles. Furthermore, these CDDs could be adapted for use with other drugs in various therapeutic applications.

## Figures and Tables

**Table 1:** The parameters of FDM 3D printing.

Parameters	Setting Conditions	
	PLA Filament	PVA Filament
Extrusion temperature	215 °C	195 °C
Diameter of filament	1.75 ± 0.05 mm	1.75 ± 0.05 mm
Nozzle diameter	0.4 mm	0.4 mm
Speed while printing	45 mm/s	45 mm/s
Speed while traveling	180 mm/s	180 mm/s
Support material	No	No
Bed temperature	60 °C	60 °C
Infill density	100%	100%
Infill pattern	Rectilinear	Rectilinear

**Table 2:** The weight variation and dimensions of the PTDs.

Samples	Height (*n* = 20) (mm)	Diameter (*n* = 20) (mm)	Hole Width (*n* = 20) (mm)	Weight (*n* = 20) (mg)
Base (PVA)	5.10 ± 0.04	12.08 ± 0.04	9.94 ± 0.06	301.54 ± 1.13
Base (PLA)	5.01 ± 0.03	11.92 ± 0.02	9.84 ± 0.03	281.57 ± 0.54
Cap 4 mm (PLA)	3.99 ± 0.01	9.93 ± 0.03	4.01 ± 0.04	182.57 ± 0.67
Cap 5 mm (PLA)	3.95 ± 0.02	9.96 ± 0.05	5.05 ± 0.05	173.17 ± 0.61
Cap 6 mm (PLA)	3.97 ± 0.04	9.95 ± 0.03	6.07 ± 0.08	161.10 ± 0.99
Cap 4 mm (PVA)	3.99 ± 0.08	10.00 ± 0.03	4.07 ± 0.06	186.78 ± 2.66
Cap 5 mm (PVA)	4.01 ± 0.03	9.95 ± 0.05	5.05 ± 0.03	179.28 ± 0.61
Cap 6 mm (PVA)	4.08 ± 0.03	9.89 ± 0.07	6.03 ± 0.04	166.86 ± 1.12
Plug 4 mm (PVA)	1.40 ± 0.02	4.06 ± 0.05	-	27.13 ± 0.34
Plug 5 mm (PVA)	1.35 ± 0.04	5.02 ± 0.06	-	36.21 ± 0.22
Plug 6 mm (PVA)	1.36 ± 0.04	6.02 ± 0.09	-	46.29 ± 0.40

**Table 3:** The weight variation and dimensions of the PGDs.

Samples	Height (*n* = 20) (mm)	Diameter (*n* = 20) (mm)	Hole Width (*n* = 20) (mm)	Weight (*n* = 20) (mg)
PGDs-L of 2 holes	6.09 ± 0.02	11.94 ± 0.04	3.80 ± 0.04	580.70 ± 1.87
PGDs-L of 3 holes	6.04 ± 0.03	11.97 ± 0.03	3.86 ± 0.05	491.39 ± 1.84
PGDs-L of 4 holes	6.08 ± 0.02	11.98 ± 0.03	3.85 ± 0.04	444.00 ± 1.98
PGDs-L of 5 holes	6.05 ± 0.01	11.97 ± 0.02	3.82 ± 0.04	401.90 ± 1.17
PGDs-V of 2 holes	6.13 ± 0.06	12.03 ± 0.09	3.95 ± 0.04	605.17 ± 1.07
PGDs-V of 3 holes	6.12 ± 0.06	12.04 ± 0.08	3.92 ± 0.04	535.62 ± 1.35
PGDs-V of 4 holes	6.18 ± 0.05	12.01 ± 0.12	3.95 ± 0.04	474.97 ± 2.02
PGDs-V of 5 holes	6.17 ± 0.03	12.06 ± 0.07	3.92 ± 0.03	427.00 ± 1.95

**Table 4:** The weight variation in prednisolone-loaded CDDs, prednisolone-loaded CDDs with enteric coating, and weight of enteric coating.

Samples	Weight of CDD-Loaded Prednisolone (mg)	Weight of CDD-Loaded Prednisolone with Enteric Coating (mg)	Weight of Enteric Coating (mg)
PTDs-LV of 4 mm	632.20 ± 1.92	661.40 ± 2.30	29.20 ± 1.30
PTDs-LV of 5 mm	630.00 ± 1.00	660.60 ± 1.30	28.80 ± 1.78
PTDs-LV of 6 mm	629.30 ± 1.09	658.80 ± 2.28	29.60 ± 1.96
PTDs-V of 4 mm	609.80 ± 0.83	639.00 ± 1.41	29.20 ± 1.92
PTDs-V of 5 mm	606.20 ± 2.28	636.00 ± 3.08	29.80 ± 1.79
PTDs-V of 6 mm	602.20 ± 1.12	631.80 ± 2.77	29.60 ± 1.63
PGDs-L of 2 holes	715.00 ± 2.23	738.40 ± 2.07	23.40 ± 1.20
PGDs-L of 3 holes	678.20 ± 2.06	702.80 ± 1.60	24.60 ± 1.51
PGDs-L of 4 holes	652.75 ± 1.64	680.60 ± 2.91	24.20 ± 0.50
PGDs-L of 5 holes	658.40 ± 2.18	683.20 ± 2.72	24.80 ± 2.06
PGDs-V of 2 holes	734.80 ± 2.94	759.00 ± 2.29	24.20 ± 2.28
PGDs-V of 3 holes	721.80 ± 1.71	745.20 ± 1.60	23.40 ± 1.30
PGDs-V of 4 holes	712.20 ± 2.58	736.00 ± 2.51	23.80 ± 1.78
PGDs-V of 5 holes	694.80 ± 2.32	719.40 ± 1.15	24.60 ± 1.30

(PTDs-LV) devices printed using PLA filament for the base and cap with PVA filament for the plug, and (PTDs-V) devices printed entirely using PVA filament for the base, cap, and plug.

**Table 5:** Analytical evaluation parameters of UV spectrophotometric method for prednisolone quantification.

Parameters	Results
Linearity	
Concentration range (µg/mL)	1.87–40
R^2^	0.9997
Slope of calibration curve	0.0116
Intercept of calibration curve	0.0013
Accuracy (%Recovery)	
15 µg/mL	100.38 ± 0.47
30 µg/mL	99.66 ± 1.00
40 µg/mL	99.40 ± 0.81
Precision (%RSD)	
15 µg/mL	0.46
30 µg/mL	1.00
40 µg/mL	0.81

**Table 6:** Drug release kinetic of prednisolone-loaded CDDs.

Samples	Zero-Order (R^2^)	First-Order (R^2^)	Higuchi (R^2^)
PTDs-LV of 4 mm	0.9098	0.9827 *	0.9519
PTDs-LV of 5 mm	0.9419	0.9905 *	0.9696
PTDs-LV of 6 mm	0.9021	0.9980 *	0.9558
PTDs-V of 4 mm	0.8471	0.9992 *	0.9433
PTDs-V of 5 mm	0.8830	0.9882 *	0.9017
PTDs-V of 6 mm	0.8197	0.9987 *	0.8423
PGDs-L of 2 holes	0.9867 *	0.8948	0.8961
PGDs-L of 3 holes	0.9837 *	0.8933	0.8930
PGDs-L of 4 holes	0.9778 *	0.8513	0.8634
PGDs-L of 5 holes	0.9687 *	0.8341	0.8133
PGDs-V of 2 holes	0.9612 *	0.7832	0.9415
PGDs-V of 3 holes	0.9902 *	0.8053	0.9790
PGDs-V of 4 holes	0.9971	0.8557	0.9977 *
PGDs-V of 5 holes	0.9952	0.8419	0.9969 *

(PTDs-LV) devices printed using PLA filament for the base and cap with PVA filament for the plug, and (PTDs-V) devices printed entirely using PVA filament for the base, cap, and plug. * Indicates the best-fit kinetic model.

## Data Availability

Data are contained within the article.

## Supplementary Materials

The following supporting information can be downloaded at: https://www.mdpi.com/article/10.3390/pharmaceutics18091185/s1, Figure S1: UV wavelength scanning spectrum of prednisolone. Figure S2: UV wavelength scanning spectrum of PVP. Figure S3: UV wavelength scanning spectrum of Eudragit^®^ S100. Figure S4: UV wavelength scanning spectrum of simulated colonic medium (pH 7.4). Figure S5: UV wavelength scanning spectrum of PVA filament. Figure S6: Calibration curve of prednisolone in simulated colonic medium (pH 7.4) at 246 nm. Each point represents the mean absorbance of triplicate measurements (*n* = 3).

## Abbreviations

The following abbreviations are used in this manuscript:

IBD	Inflammatory bowel disease
BCS	Biopharmaceutics classification system
CTDDSs	Colon-targeted drug delivery systems
3D	Three-dimensional
4D	Four-dimensional
FDM	Fused deposition modeling
HME	Hot melt extrusion
PVA	Polyvinyl alcohol
PLA	Polylactic acid
CDDs	3D-printed drug-containing devices
GPO	Government pharmaceutical organization
PVP	Polyvinylpyrrolidone
Na_2_HPO_4_ · 7 H_2_O	Sodium phosphate dibasic heptahydrate
KH_2_PO_4_	Potassium phosphate monobasic
NaCl	Sodium chloride
KCl	Potassium chloride
HCl	Hydrochloric acid fuming 37%
NaOH	Sodium hydroxide
PTDs	Devices for prednisolone tablets
PGDs	Devices for prednisolone gel
PTDs-LV	PLA filament for the base and cap with PVA filament for the plug
PTDs-V	PVA filament for the base, cap, and plug
PGDs-L	The devices produced using PLA filament
PGDs-V	The devices produced using PVA filament
SEM	Scanning electron microscope
SD	Standard deviation
ANOVA	One-way analysis of variance
LOD	limit of detection
LOQ	limit of quantification
RSD	Relative standard deviation
