# Supplementary material for: Innovative 3D-Printed Devices with Modified Prednisolone Release for Colon-Targeted Drug Delivery Systems
Source: Pharmaceutics. 2026 Sep 19;18(9):1185. doi: 10.3390/pharmaceutics18091185 (PMC13610189; doi:10.3390/pharmaceutics18091185)
Supplement: Supplementary file 1 [file pharmaceutics-18-01185-s001.zip › pharmaceutics-4529644-supplementary.pdf]

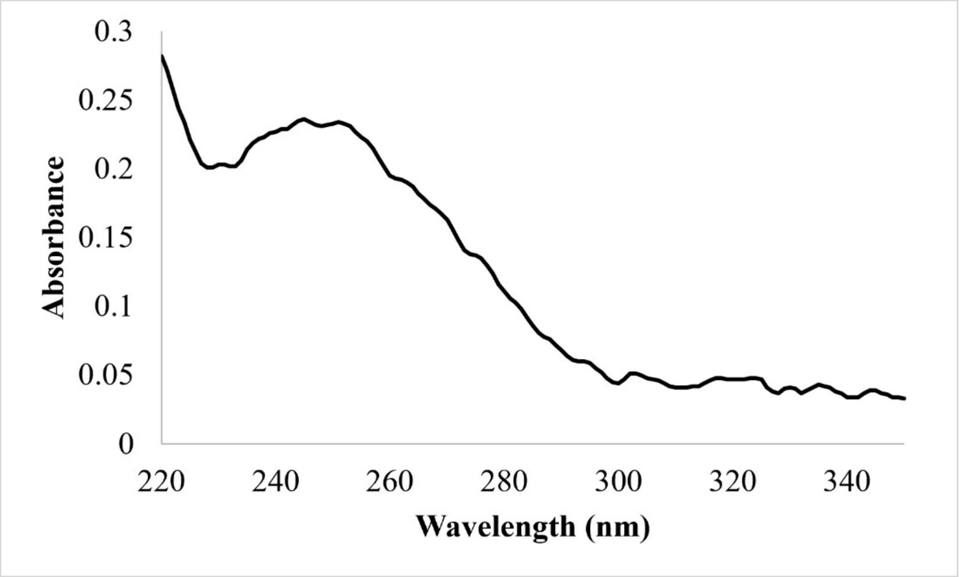

Supplementary Figure S1. UV wavelength scanning spectrum of prednisolone.

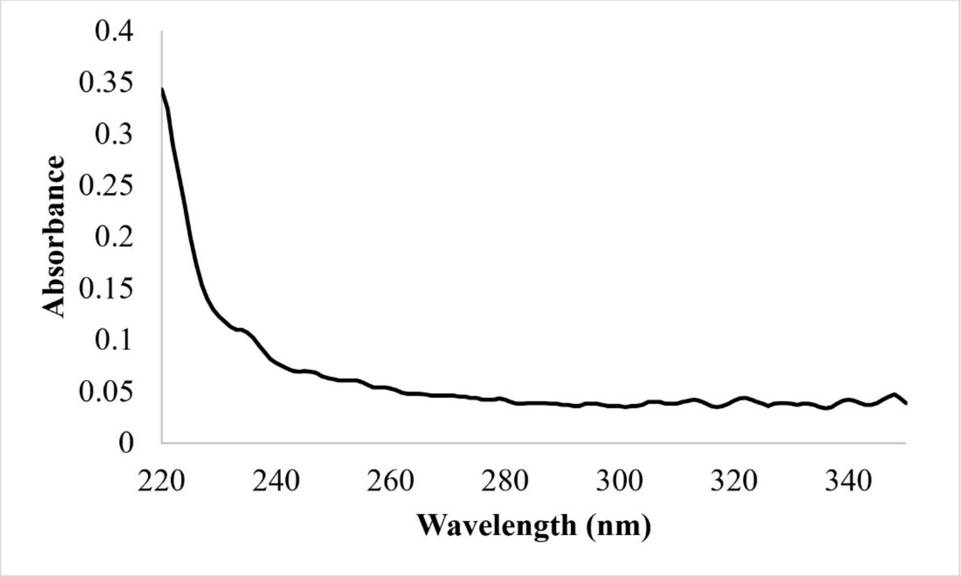

Supplementary Figure S2. UV wavelength scanning spectrum of PVP.

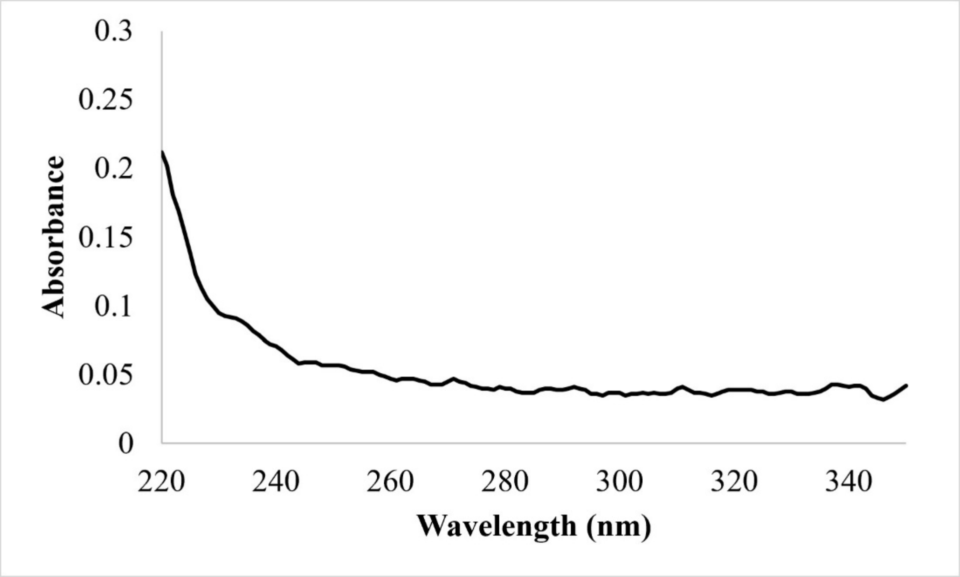

Supplementary Figure S3. UV wavelength scanning spectrum of Eudragit® S100.

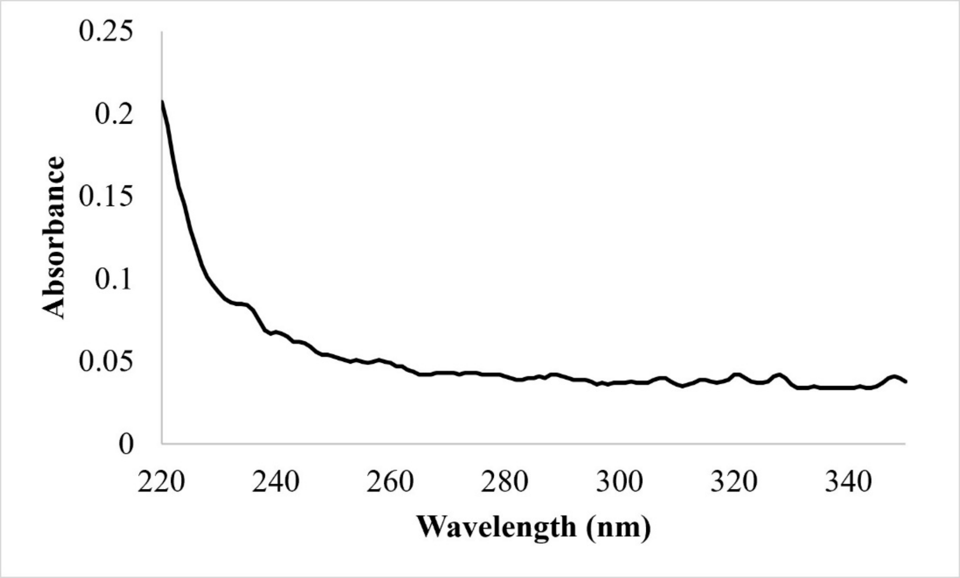

Supplementary Figure S4. UV wavelength scanning spectrum of simulated colonic medium (pH 7.4).

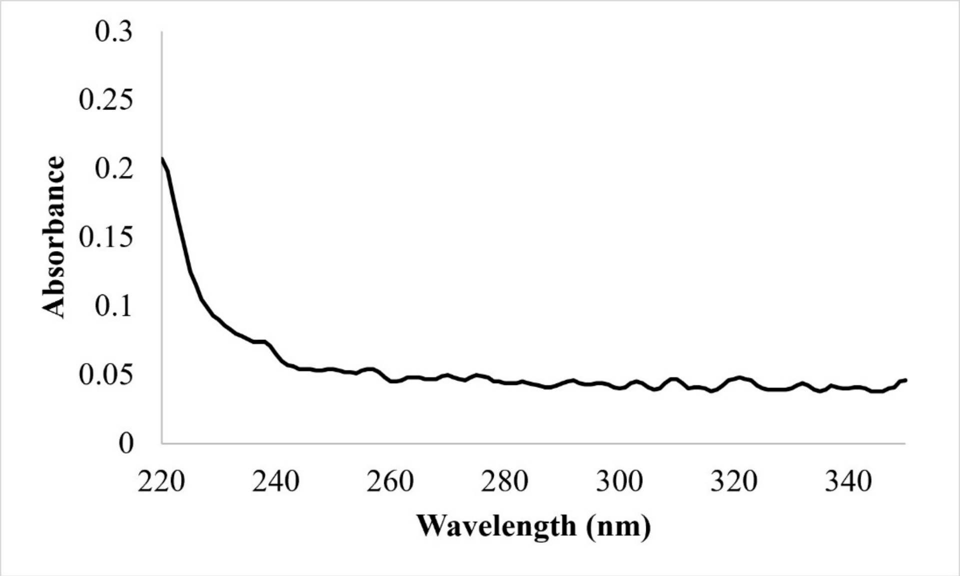

Supplementary Figure S5. UV wavelength scanning spectrum of PVA filament.

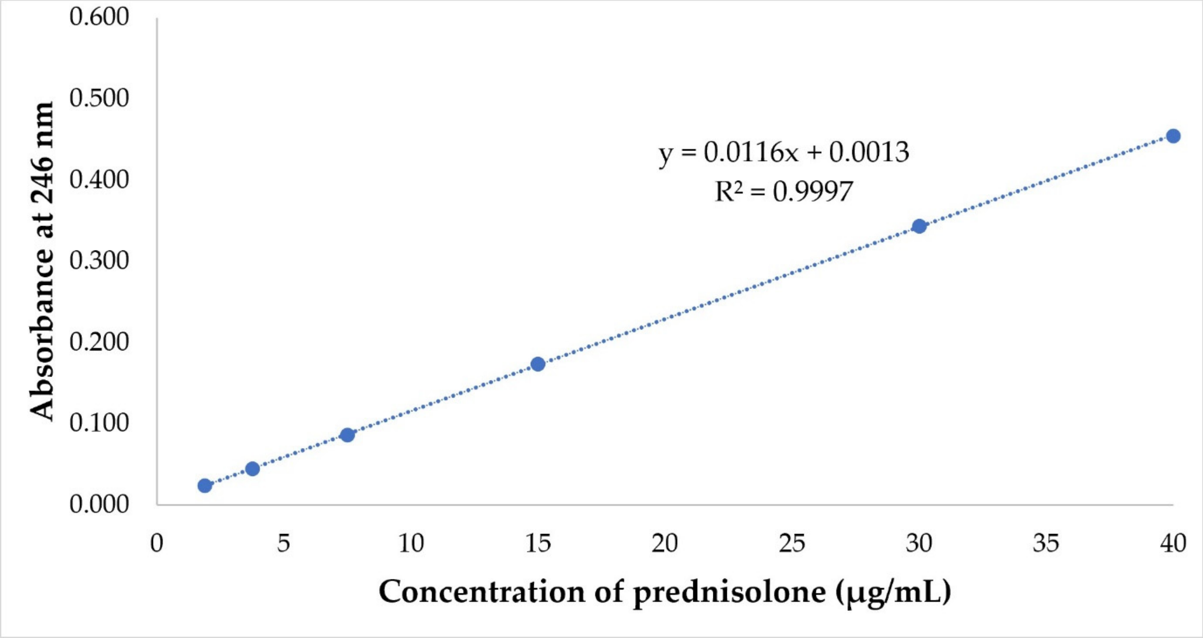

Supplementary Figure S6. Calibration curve of prednisolone in simulated colonic medium (pH 7.4) at 246 nm. Each point represents the mean absorbance of triplicate measurements (*n* = 3).
